# Spray cryotherapy for actively bleeding refractory gastric arteriovenous malformations results in immediate and durable hemostasis

**DOI:** 10.1055/a-2587-8605

**Published:** 2025-05-19

**Authors:** Pavithra Ramakrishnan, Mohammad Bilal, Brian Hanson, Susan Lou

**Affiliations:** 1 14400 Department of Internal Medicine, University of Minnesota Medical Center, Minneapolis, Minnesota, United States; 2 20040 Division of Gastroenterology and Hepatology, Minneapolis Veterans Affairs Health Care System, Minneapolis, Minnesota, United States

**Fig. 1:**
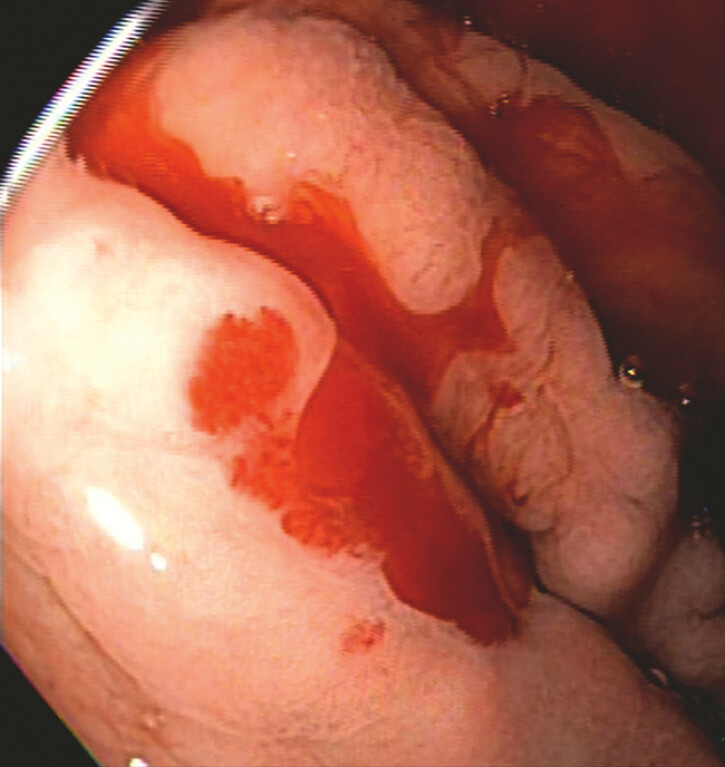
Arteriovenous malformations in the gastric body with visible oozing.

Cryotherapy utilizes targeted freeze-thaw cycles to disrupt cellular integrity and induce cell death. Initially employed for the treatment of mucosal and submucosal neoplasms, its therapeutic applications have since expanded. In this case, we describe the management of a 71-year-old man with refractory gastrointestinal bleeding. Endoscopy revealed multiple active bleeding arteriovenous malformations (AVMs), ranging from 3 to 5 mm, located in the gastric body ( Fig. 1 ). Given the persistent nature of bleeding, the decision was made to treat these AVMs with spray cryotherapy.

Endoscopic visualization confirmed the target sites for ablation. A 20 Fr dual-channel decompression tube was inserted over a guidewire into the gastric body to facilitate active venting and prevent barotrauma. After the tube was in place, the endoscope was reinserted, and the ablation catheter was advanced through the working channel. Six AVM sites were ablated, as shown in Video 1 . For each lesion, liquid nitrogen cryogen was applied for 20 s following the formation of a visible frost effect ( Video 1 ). The ablated area was then allowed to thaw for 60 s before the cycle was repeated. Each lesion received 1–2 cycles of cryotherapy.

Application of cryotherapy in the treatment of actively bleeding gastrointestinal arteriovenous malformations: an instructional video.Video 1

**Fig. 2:**
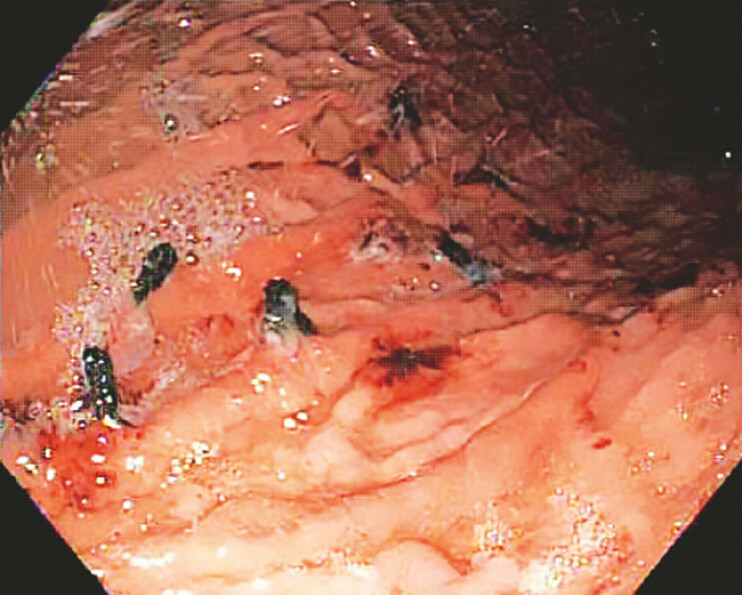
Appearance of mucosa immediately following cryotherapy.

The procedure was completed without any immediate complications. Postprocedural retroflexion of the endoscope revealed nonbleeding beefy red appearing gastric mucosa ( Fig. 2 ). This case demonstrates the expanding use of cryotherapy in treating gastrointestinal arteriovenous malformations, offering an effective and durable solution for managing refractory bleeding. This approach highlights the evolving role of cryotherapy in endoscopic interventions for complex gastrointestinal pathologies.

Endoscopy_UCTN_Code_TTT_1AO_2AD

